# Mastocytosis and systemic sclerosis: a clinical association

**DOI:** 10.1186/s12948-016-0050-3

**Published:** 2016-10-11

**Authors:** Gianluca Bagnato, William Neal Roberts, Davide Sciortino, Donatella Sangari, Santa Cirmi, Roneka L. Ravenell, Michele Navarra, Gianfilippo Bagnato, Sebastiano Gangemi

**Affiliations:** 1 Division of Rheumatology, Department of Clinical and Experimental Medicine, University of Messina, Messina, Italy; 2 Division of Rheumatology, Department of Medicine, University of Louisville, Louisville, KY USA; 3 Department of Chemical, Biological, Pharmaceutical and Environmental Sciences, University of Messina, Messina, Italy; 4 School and Division of Allergy and Clinical Immunology, Department of Clinical and Experimental Medicine, University of Messina, Messina, Italy; 5 Institute of Applied Sciences and Intelligent Systems (ISASI), Pozzuoli, NA Italy

**Keywords:** Mastocytosis, Systemic sclerosis, Mastcells, Fibrosis

## Abstract

**Background:**

Systemic sclerosis (SSc) is a complex autoimmune disease characterized by vascular alterations and autoimmune activation leading to widespread organ fibrosis. At the early stage of disease when organ involvement and extent of disease are emerging, mast cells may have some role, as implied by both symptoms and histologic evidence.

**Case presentation:**

A female patient diagnosed with cutaneous mastocytosis experienced the onset of systemic sclerosis after 15 years followed by the switch of mastocytosis to the systemic phenotype. A literature review on the evidences related to mast-cells activation in systemic sclerosis is presented below.

**Conclusions:**

For clinicians, more attention must be paid to the potential association between systemic sclerosis and cancer. This case suggests that a proliferative disease in the mast cell compartment—though representing a rare association—may not be completely unexpected in SSc and perhaps excess mast cell activity can serve a pathogenic role in promoting fibrotic disease.

## Background

Systemic sclerosis (SSc) is a connective tissue disorder characterized by autoimmune activation and endothelial dysfunction leading to fibrotic changes of the skin and internal organs [1]. The clinical presentation of this disease is highly heterogeneous. In addition, prediction of the ultimate pattern of specific internal organ involvement, as well as the development of overlap features, is difficult at the time of the diagnosis [2]. Early in the course, patients with SSc skin disease report pruritus, being described frequently as the most bothersome symptom [3–5]. Among 400 patients, 45 % reported pruritus with a disease duration of at least 1 year, increasing to 69 % when considering only patients with 1–2 years of disease duration [6]. Lip biopsy in very early stages of SSc reveals that mast cell infiltration starts before the disease acquires definite features [7]. Biopsy of involved skin of SSc patients confirms that mast cells (MC) reside in proximity of fibroblasts, produce TGF-β and the number of degranulated cells is particularly increased [8]. More specifically, the MCT (tryptase-positive, chymase-negative) are more prominent in SSc lesional skin compared to MCTC (tryptase-positive, chymase-positive) type of mast cell that represents the normal population of mast cell in healthy subjects [9].

Nevertheless, it remains unclear whether the switch toward a specific population of MC favours fibroblast proliferation through the release of tryptase, but it might. The early phase of SSC includes interstitial oedema and pruritus, the corticosteroid responsive so-called “puffy hands” phase of SSc clinically. Mast cell mediator release may promote the early phase of interstitial oedema and pruritus through histamine and the processing of big-endothelin in endothelium through chymase, as evidenced in rat lungs. On the other hand, mast cells can also counteract collagen deposition through matrix metalloproteinase activation [10].

Although mastocytosis is rare in any setting, the incidence of malignancy in general in SSc is increased compared to the general population (pooled standardized incidence ration 1.41 for all cancers). The incidence of cancer is higher within the first 12 months after the initial SSc diagnosis [11]. In population-based studies of SSc patients, it has been reported that the hematologic malignancies are primarily non-Hodgkin lymphomas and leukemias [12], and in female subjects hematologic cancer is more frequent [13]. Consequently, a proliferative disease in the mast cell compartment—though representing a rare association—may not be completely unexpected in SSc. In this report, we call attention to a case of patient who developed SSc after, rather than before, a diagnosis of cutaneous mastocytosis, and in whom the mastocytosis became more extensive after the SSc became manifest.

## Case presentation

A 36 year-old woman presented with complaints of dysphagia, fatigue, widespread pain and muscle weakness localized mainly at the inferior limbs. Fifteen years prior, she was diagnosed with cutaneous mastocytosis, confirmed by skin biopsy. Over the 6 months, she noticed worsening of Raynaud’s phenomenon and thickening of the skin over the hands and feet. She underwent videocapillaroscopy revealing an active scleroderma pattern and was diagnosed with systemic sclerosis. Laboratory test showed ANA positivity with high titers (1:2560, granular pattern) and anti-SSa. Four months later, she was admitted to the hospital due to worsening dysphagia, fatigue and muscle weakness.

Clinical examination revealed body temperature 36.5 °C, blood pressure 127/80 mmHg and a regular pulse of 76 beats/min. No superficial lymph adenopathy was evident. Skin thickening (Rodnan skin score 28/51) and spleen enlargement were noted, however. Laboratory tests revealed that rheumatoid factor, as well as anti-SS-B, anti-Sm, anti-Scl-70, anti-centromere, anti-Jo1 and anti-DNA antibodies were all negative. Serum levels of CK 350 U/L (n.v.197 U/L), LDH 652 U/L (n.v. 200 U/L), myoglobin (421 ng/mL n.v. 0–70 ng/mL), SGOT 47 U/L (n.v. 29 U/L), SGPT 71 U/L (n.v. 51 U/L) were elevated. The serologic HLA typing was A1, B51 and CW7 for class I and DQ7, DR11 and DR52 for class II.

Ultrasound examination of the spleen confirmed the physical examination finding. Echocardiographic examination was relevant for an increase in pulmonary arterial pressure (40 mmHg). Due to the suspicion of the onset of a systemic mastocytosis, based on the involvement of the spleen and vertebral pain, the patient underwent bone marrow biopsy, PET and total body CT. Bone marrow biopsy result confirmed an intense infiltration of mast cells triptase+ , CD25+ , CD117+ , CD123+ , CD45RB/LCA+ , CD68+ , CD14± ,CD20−, CD30−. PET scan showed thoracic and abdominal lymph nodes, spleen and sacrum uptake. CT scan detected widespread lytic lesion of the chest and vertebral bones, basilar reticulonodular infiltrates, and spleen enlargement. Electromyography was compatible with an inflammatory myopathy.

The patient started therapy with prednisone at the dose of 50 mg day, nifedipine 10 mg day, mycophenale mofetil at the dose of 2 g day and iloprost infusions 0.05 mg every day per 3 days every month. She underwent full evaluation in a heamotologic center located in a different hospital, and received zoledronic acid for osteolytic lesions. After 2 months, the patient’s myalgias and fatigue improved, as confirmed by serology, as well as her vertebral pain. The addition of a chemotherapeutic agent for systemic mastocytosis is now under consideration.

## Conclusions

A previous case series study described this rare association in which systemic sclerosis precedes the onset of cutaneous mastocytosis [14], perhaps suggesting some underlying stimulation of the mast cell compartment as a feature of the SSc. The investigators reported two cases of skin lesions associated with mast-cell infiltrates compatible with the diagnosis of cutaneous mastocytosis at 30 and 6 years after the onset of SSc. In contrast, in our case, the initial cutaneous mastocytosis has an interplay with SSc in which cutaneous mastocytosis precedes the onset of systemic sclerosis, perhaps suggesting that excess mast cell activity can serve a pathogenic role in promoting fibrotic disease. The onset of SSc in this case in turn was subsequently accompanied by the progression of the cutaneous form of mastocytosis into the widespread systemic phenotype. This case therefore raises the possibility that, the link between mast cell activity and fibrotic disease may ultimately be understood as a bidirectional one, or a feedback loop.

Several lines of evidence support the involvement of mast-cells in the pathogenesis of SSc [15, 16]: in the tight skin mouse model of SSc the proliferation and activation of mast-cells leads to augmentation of fibrosis; an increase of dermal mast cell density is typical of the early phase of the disease [17]; mast cells are involved in the development of interstitial oedema [18]; the degranulation of mast-cells is considered one of the mechanisms leading to TGF-β secretion in SSc [19]. Indeed, mast cells granules contain several profibrotic moelcules, such as TGF-β itself and also PDGF and VEGF, plus other molecules that favour myofibroblast transdiferrentiation, such as IL-4 and GM-CSF [20].

Of note, among the chemotherapeutic agents available for the treatment of mastocytosis, a small tyrosine kinase inhibitor, imatinib mesylate, has indications for specific forms of this malignancy (without the D816 V c-Kit mutation or with c-Kit mutational status unknown) [21] though it failed in SSC [22]. Similarly, in SSc another related tyrosine kinase inhibitor, nintedanib, has gained increasing attention as a potential therapeutic agent in interstitial pulmonary fibrosis. Promising results have been reported in animal models [23] and in clinical trials related to skin fibrosis and lung function [24]. Nintedanib is now in clinical trial for ILD due to SSc. If tyrosine kinase inhibitors are found to play a role in therapeutic regimens aimed at fibrotic diseases such as idiopathic pulmonary fibrosis (IPF) and systemic sclerosis associated interstitial lung disease (SSc–ILD), it may be that they work in part by inhibition of slower, chronic, non-anaphylactic transdegraulation of mast cells, among other mechanisms.

## Abbreviations

SSc: systemic sclerosis
MC: mastcells
MCT: mast cells tryptase-positive chymase-negative
MCTC: mast cells tryptase-positive chymase-positive
ANA: antinuclear antibodies
anti-SS-A: anti-Sjögren’s-syndrome-related antigen A
anti-SS-B: anti-Sjögren’s-syndrome-related antigen B
anti-Sm: anti-Smith antibodies
anti-Scl-70: anti-topoisomerase I antibodies
anti-Jo1: anti-intracellular enzyme histidyl tRNA synthetase antibodies
CK: creatine kinase
LDH: lactate dehydrogenase
SGOT: serum glutamic-oxaloacetic transaminase
SGPT: serum glutamic-pyruvic transaminase
HLA: human leukocyte antigen
CT: computerized tomography
CD: cluster of differentiation
PET: positron emission tomography
TGF-β: transforming growth factor β
PDGF: platelet-derived growth factor
VEGF: vascular endothelial growth factor
IL-4: interleukin-4
GM-CSF: granulocyte-macrophage colony-stimulating factor
IPF: idiopathic pulmonary fibrosis
SSc-ILD: systemic sclerosis associated interstitial lung disease
c-kit: tyrosine-protein kinase kit
